# Sprouted Wheat Improves Liver Metabolism and Inflammation in T2DM Mice: 16S rRNA Gene Sequence, Metabolomics and Network Pharmacology Joint Analysis

**DOI:** 10.3390/foods15061027

**Published:** 2026-03-15

**Authors:** Xue Gao, Qifang Guo, Peihua Li, Yanquan Mu, Huajing Gao, Qinglin Qu, Jiaqi Liu, Fan Yang, Dapeng Li, Feng Li, Xintong Tan

**Affiliations:** 1 Key Laboratory of Food Nutrition and Human Health in Universities of Shandong, College of Food Science and Engineering, Shandong Agricultural University, Tai’an 271018, China; gaox120959@163.com (X.G.); liph010520@163.com (P.L.); myq2571828268@163.com (Y.M.); myszghj@163.com (H.G.); quqinglin2024@163.com (Q.Q.); sdtaljq@163.com (J.L.); fanen111@163.com (F.Y.); lidapengsdau@163.com (D.L.); 2 National Key Laboratory of Wheat Breeding, College of Agronomy, Shandong Agricultural University, Tai’an 271018, China; gqf@sdau.edu.cn

**Keywords:** type 2 diabetes mellitus, sprouted wheat, gut microbiota, metabolic disorders, inflammation, network pharmacology

## Abstract

Type 2 diabetes mellitus (T2DM) has become a global metabolic disorder, and sprouted wheat (SW) exhibits potential for alleviating metabolic syndromes, although its mechanism remains unclear. This study aimed to investigate the effects and underlying mechanisms of SW on T2DM using a high−fat diet−induced T2DM mouse model. SW intervention significantly improved glycolipid metabolism disorders (*p* < 0.05), attenuated hepatic mitochondrial injury (*p* < 0.05) and maintained hepatic homeostasis. SW also reshaped the gut microbiota structure and inhibited the TLR4/NF−κB inflammatory pathway (*p* < 0.05). Untargeted metabolomics combined with network pharmacology identified five key functional metabolites and four core targets involved in the protective effects of SW. Germination optimized the nutritional composition of wheat, and SW regulated the microbe–liver axis through a multi−component, multi−target and multi-pathway mode. These results reveal the mechanism of SW in improving T2DM−related metabolic disorders and provide experimental support for its application. In the future, SW can be further developed as a dietary nutritional supplement for the prevention and adjuvant treatment of metabolic diseases.

## 1. Introduction

Diabetes mellitus is a prevalent chronic metabolic disorder accompanied by severe complications, which severely reduces patients’ quality of life and shortens life expectancy [1]. According to the World Health Organization, more than 500 million people worldwide are affected by diabetes, among which T2DM accounts for 90–95% of all cases [2]. The incidence of T2DM is closely associated with long−term high−calorie diets, which overload the metabolic organs, trigger systemic metabolic disorders and induce chronic low−grade inflammation [3]. Notably, unhealthy dietary patterns disrupt intestinal homeostasis, impair intestinal barrier integrity, promote the translocation of harmful metabolites into the liver and trigger sustained inflammatory responses and insulin resistance [4]. Current interventions for T2DM mainly include pharmacological therapy, physical exercise and dietary regulation. However, long−term administration of conventional hypoglycemic drugs frequently causes side effects such as gastrointestinal discomfort and hypoglycemia, and may also increase the metabolic burden on the kidneys in patients with advanced diabetes [5]. Therefore, the development of safe, effective and non-pharmacological intervention strategies is of great clinical significance. As a mild and sustainable approach, dietary intervention plays an irreplaceable role in the prevention and control of T2DM [6].

Wheat, one of the most important staple crops worldwide, provides abundant nutrients including carbohydrates, proteins and dietary fiber [7,8]. International dietary guidelines consistently recommend that increased whole−grain intake effectively reduces the risk of chronic diseases including diabetes, obesity and non−alcoholic fatty liver disease [9]. However, refined wheat flour, which dominates modern dietary patterns, is processed to remove bran and germ, leaving mainly rapidly digestible starch. Such composition leads to sharp postprandial blood glucose fluctuations and disrupts glucose homeostasis [10]. This composition causes rapid postprandial blood glucose elevation, thereby disrupting glucose homeostasis [11]. Improving the nutritional quality and health benefits of wheat−based products is therefore paramount [12]. Germination is a natural, eco-friendly and low−cost processing method that effectively improves the nutritional value and bioactive component profiles of cereal grains [13,14]. For example, germinated brown rice and black rice have been reported to regulate blood lipid and glucose levels and modulate the expression of lipid metabolism−related enzymes in T2DM models [15]. Emerging evidence indicates that sprouted wheat (SW) exhibits favorable metabolic regulatory effects and helps maintain glucose homeostasis, representing a promising functional food ingredient [16,17]. Current evidence indicates that germinated grains can ameliorate glucose and lipid metabolic disorders, relieve chronic inflammation and modulate gut microbiota composition in metabolic diseases [18]. The gut−liver axis has been recognized as a pivotal route through which dietary interventions improve T2DM by regulating microbial metabolites, intestinal barrier integrity, hepatic insulin sensitivity and inflammatory reactions [19]. Nevertheless, whether sprouted wheat exerts protective effects against metabolic and inflammatory disturbances in T2DM mice via mediating the gut−liver axis remains to be further elucidated.

Accordingly, this study established a chronic high−fat diet−induced T2DM mouse model and used a comprehensive strategy integrating microbiome analysis, molecular biology, non−targeted metabolomics, network pharmacology and molecular docking. Intestinal microbiome analysis was applied to characterize SW−mediated alterations in gut microbial composition and function. Metabolomics was used to identify key bioactive components enriched during germination. Network pharmacology and molecular docking were further performed to identify potential targets and signaling pathways. In vivo experiments validated the regulatory effects of SW on insulin and inflammatory pathways. Collectively, this multi-dimensional systematic study confirmed that SW alleviates T2DM by reshaping gut microbiota and regulating downstream metabolic and inflammatory pathways. This work clarifies the molecular mechanism of SW in improving T2DM and provides a novel nutritional intervention strategy for the prevention and treatment of metabolic disorders using germinated cereal grains.

## 2. Materials and Methods

### 2.1. Reagents and Materials

High-purity acetonitrile, methanol and formic acid were respectively provided by Yongda Chemical Reagents (Tianjin, China), Fisher Chemical (Beijing, China) and Kermel (Tianjin, China).

### 2.2. Preparation of Sprouted Wheat

Wheat cultivar Shannong 28 (*Triticum aestivum* L.) was used in this study. The wheat was cultivated in Shandong Province, China, and harvested in 2024. Prior to germination, the wheat grains were stored in a dry, well−ventilated environment at 20 ± 2 °C and 45 ± 5% relative humidity (RH) for 1 month to ensure stable dormancy and avoid mold growth. Before germination, seed vigor was not separately evaluated, but uniform wheat grains with an intact seed coat, full shape and no damage or mildew were manually selected to ensure consistent physiological quality and stable germination performance. To ensure the stability of germination, sample safety and nutrient accumulation, we referred to previous studies and selected a germination condition of 15 °C for 3 days [20,21]. The specific operation is as follows: the wheat samples were disinfected and washed with distilled water and soaked for 12 h. The soaked wheat was placed in a germination dish, covered with wet gauze on the top, and then sprouted in the dark at a relative humidity of 80% and a temperature of 15 °C in an incubator for 3 days; then, it was placed in an oven at 37 °C and dried; finally, it was thoroughly ground and sieved through an 80−mesh sieve. To ensure biochemical stability, all analytical determinations were conducted within 24 h after germination.

### 2.3. Components Analysis and Structural Characterization of Sprouted Wheat

(1) Components Analysis

The starch content in wheat was determined using the total starch kit (Solébo, Beijing, China). The samples were digested with concentrated sulfuric acid and the protein content was determined by an automatic Kjeldahl nitrogen analyzer (Hanon, Jinan, China). The determination of fat and dietary fiber was carried out according to the method of AOAC [22]. The determination methods for total polyphenols, total flavonoids and antioxidant activity were carried out according to the scheme described in the literature [23].

(2) Structural Characterization

**Figure 1:**
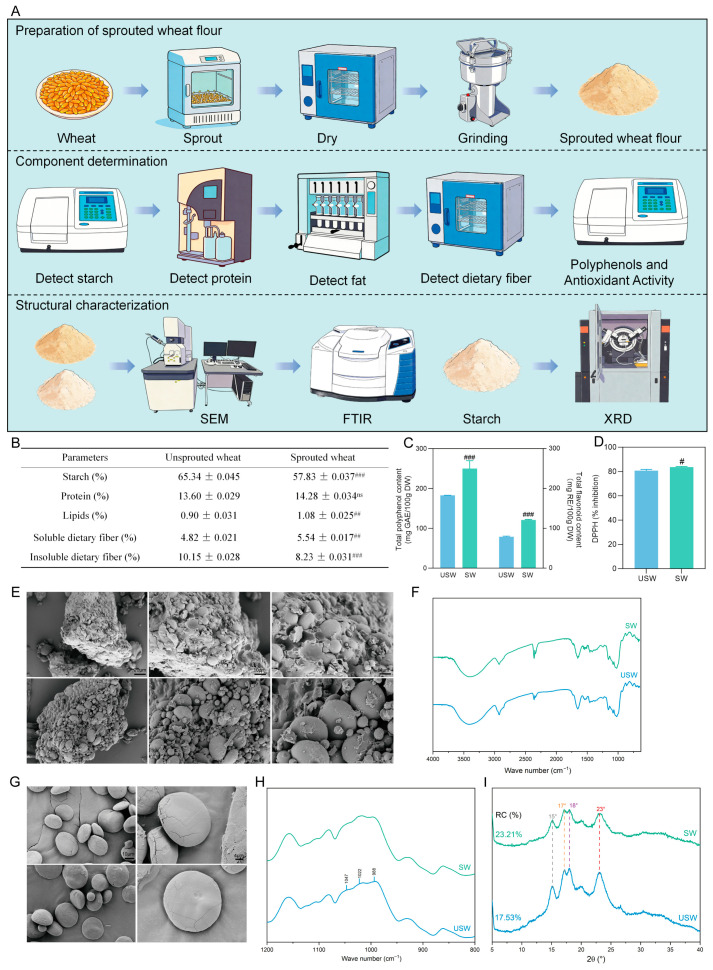
Impact of germination on the nutritional components and structural characterization of wheat. (**A**) The preparation and analysis process of SW. (**B**) The contents of starch, protein, fat, soluble dietary fiber and insoluble dietary fiber in USW and SW. (**C**) The contents of total polyphenols and total flavonoids. (**D**) DPPH radical scavenging abilities. (**E**,**F**) SEM and FTIR. (**G**–**I**) SEM, FTIR and XRD of the starch in USW and SW. Dashed lines represent the characteristic diffraction angles (°, 2θ) of the sample. *n* = 3–6. ^#^ *p* < 0.05, ^##^ *p* < 0.01, ^###^ *p* < 0.001 versus USW, ns: no significant difference.

The extraction steps of starch are based on previous studies [24]. To systematically investigate the effects of the germination process on the characteristics of wheat and starch, multi−dimensional structural characterization analysis needs to be conducted on the wheat samples and the extracted starch samples. Among them, the surface morphology and structural changes in the samples were analyzed using scanning electron microscopy (SEM, Carl Zeiss, Guangzhou, China), fourier transform infrared spectroscopy (FTIS, thermo fisher, Shanghai, China) and X−ray diffraction (XRD, Rigaku Corporation, Beijing, China) techniques. Detailed data can be found in the Supplementary Materials. The basic experimental steps are shown in Figure 1A.

### 2.4. Ultra Performance Liquid Chromatography−Mass Spectrometry (UPLC−MS)

**Figure 2:**
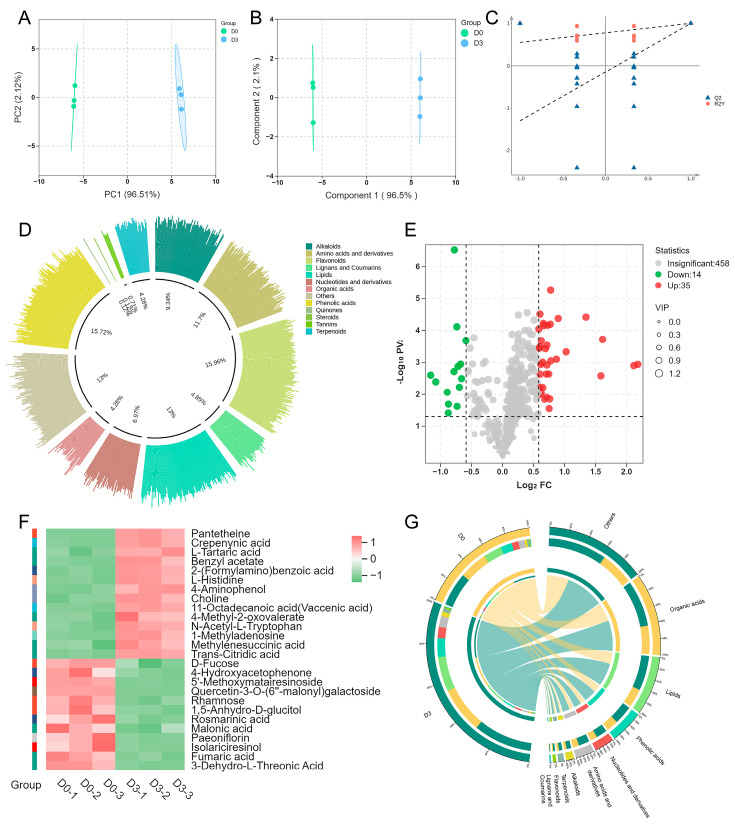
Untargeted metabolomics analysis of SW. (**A**) PCA analysis diagrams of the two groups of wheat. (**B**) The OPLS−DA analysis plots of the two groups of wheat. (**C**) Data model replacement test graph. Dashed line: regression line of the permutation test. (**D**) Classification of 507 metabolites of two groups of wheat. (**E**) Volcano plot of differentially expressed metabolites in the SW group compared with the USW group. (**F**) Differential metabolite heatmap. (**G**) Enrichment chord diagram of differential metabolites. Data are presented as mean ± SEM, *n* = 3.

The metabolic profile of SW is mainly detected by the UPLC-MS method (Figure 2). About 100 mg each of sprouted and unsprouted wheat flour were weighed, 1.2 mL of 70% methanol solution was added, vortexed for 30 s and subjected to ultrasound for 30 min, overnight at 4 °C. After centrifugation at 11,000× *g* (relative centrifugal force, RCF) for 10 min, the supernatant was collected and filtered through a 0.22 μm organic phase filtration membrane for samples analyzed by liquid chromatography–mass spectrometry. Each sample was set up in triplicate and QC samples were added to ensure the stability of analysis. The ultra−high−performance liquid chromatograph (LC40, Shimadzu, Kyoto, Japan) was used in combination with the mass spectrometer (ZenoTOF 7600, AB SCIEX, Concord, ON, Canada) for evaluating SW and common wheat. Separation was achieved on an Agilent SB−C18 column (100 × 2.1 mm, 1.8 μm, Shanghai, China) maintained at 30 °C, with a mobile phase flow rate of 0.3 mL/min and an injection volume of 2 μL. The mobile phases consisted of 0.1% formic acid in water (A) and acetonitrile (B). The elution gradient was set as follows: 0–2 min, 95% A; 2–18 min, 5% A; 18–20 min, 95% A. Analysis was performed in both positive and negative ionization modes under optimized instrument parameters: ion spray voltage, −4500 V (negative) and 5500 V (positive); curtain gas pressure, 50 psi; nebulizing gas, 55 psi; ion source temperature, 500 °C; declustering potential, ± 100 eV; collision energy, −35 eV (negative) and 10 eV (positive); mass scanning range, *m*/*z* 50–1000 [25].

### 2.5. Experimental Animals and Grouping

Twelve−week−old C57BL/6J mice (male, weight 24–26 g) were provided in SPF (Jinan) Biotechnology Co., Ltd. (Jinan, China). They were maintained under standard conditions (ambient humidity at 50% ± 15% and temperature at 23 ± 2 °C) with free access to water and food. The Trition X−100 and immunohistochemical kits were purchased from Beijing Zhongshan Jinqiao Biotechnology Co., Ltd. (Beijing, China). All antibodies were purchased from Abways Technology (Shanghai, China).

**Figure 3:**
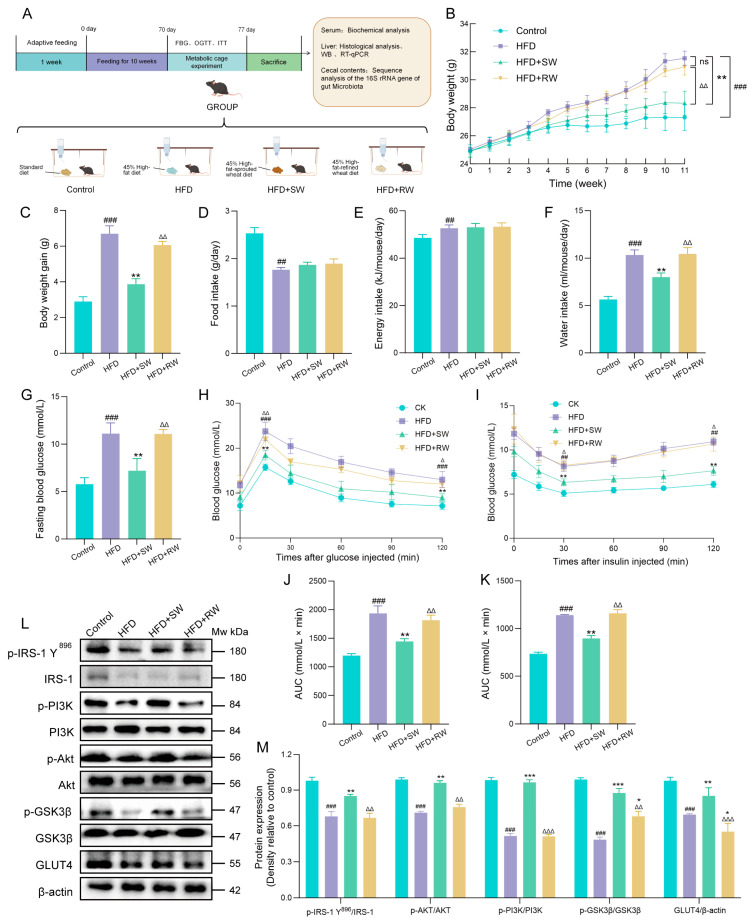
The effect of sprouted wheat on the basic indicators and insulin resistance of T2DM mice. (**A**) The timeline of the diet of each group of mice. (**B**) Body weight. (**C**) Body weight gain. (**D**) Food intake. (**E**) Energy intake. (**F**) Water intake. (**G**) Fasting blood glucose. (**H**) Oral glucose tolerance test. (**I**) Insulin tolerance test. (**J**,**K**) AUC of blood glucose. (**L**,**M**) Western blots of IRS1/PI3K/AKT/GSK3β/GLUT4 signaling. Data are presented as mean ± SEM, *n* = 3–8. ^##^ *p* < 0.01, ^###^ *p* < 0.001 versus control group, * *p* < 0.05, ** *p* < 0.01, *** *p* < 0.001 versus HFD group; ^∆^ *p* < 0.05, ^∆∆^ *p* < 0.01, ^∆∆∆^ *p* < 0.001 versus HFD+SW group, ns: no significant difference.

After 1 week of adaptation, the mice were randomly divided into four groups. This experiment involved a total of 32 mice, with 8 mice in each group. Control mice were fed a regular maintenance diet. The mice in the model group were fed a 45% high−fat diet. Mice in the HFD+SW group and the HFD+RW group were fed a 45% high−fat diet, the carbohydrate portion of which was isocalorically replaced with SW and refined flour, respectively. The HFD+RW group was primarily established to simulate the daily dietary patterns of people and evaluate the effectiveness of substituting refined staple foods with SW. The detailed grouping is shown in Figure 3A. During the experiment, the mental state, activity and diet of the mice, as well as any deaths, were observed. There were no abnormal situations during the experiment. All animal experiments followed the relevant guidelines in the UK Animals (Scientific Procedures) Act 1986 and European Union Directive 2010/63/EU. All experiments were approved by the Animal Committee of Shandong Agricultural University (Approval No.: SDAU 18−096; Approval Date: 6 July 2018).

### 2.6. Determination of Glucose Metabolism

In the 11th week of dietary intervention, an oral glucose tolerance test (OGTT) was conducted on the mice. The mice were fasted for 12 h with free access to water, followed by the oral administration of a glucose solution at a dose of 2 g/kg body weight. Blood samples were collected from the tail vein at 0, 15, 30, 60, 90 and 120 min post-administration, and blood glucose levels were measured using a Sinocare glucometer (Model: GA−3; Sinocare Inc., Changsha, China). The total glycemic response was quantified by calculating the area under the curve (AUC) for glucose concentration versus time. For the insulin tolerance test (ITT), mice were fasted for 4 h and administered 0.75 U/kg insulin via intraperitoneal injection. Blood glucose measurements were taken at specified intervals from the tail vein, and the AUC was similarly computed to evaluate the insulin-mediated glucose disposal.

### 2.7. Metabolic Cage Analysis

The mice were housed individually in the Homecage metabolic cage system for 24 h under a 12 h light–dark cycle. During this period, food intake, O_2_ consumption, CO_2_ production and spontaneous activity of the mice were recorded. Raw data were collected and processed by the Homecage system to analyze the respiratory exchange rate (RER) and energy expenditure (EE) of the mice.

### 2.8. Biochemical Analysis

The content of alanine aminotransferase (ALT), aspartate aminotransferase (AST), alkaline phosphatase (ALP), albumin (ALB), total protein (TP) and lactate dehydrogenase (LDH) in serum and the levels of triglycerides (TG), total cholesterol (TCHO), high-density lipoprotein cholesterol (HDL−C) and low−density lipoprotein cholesterol (LDL−C) were determined using an automatic biochemical analyzer (ACE, West Caldwell, NJ, USA). The test was conducted according to the instructions provided in the Zecen biological test kit (Zecen, Taizhou, China). Briefly, serum samples were centrifuged at 3000× *g* for 10 min at 4 °C to remove impurities prior to detection. All biochemical indices were tested strictly according to the instructions provided in the Zecen biological test kit (Zecen, Taizhou, China), and the detection wavelength, reaction temperature and reaction time were set in accordance with the kit specifications and the manufacturer’s operation manual of the automatic biochemical analyzer [26].

### 2.9. Histological Analysis

After the mice were euthanized, their liver tissues were removed and partially embedded in wax-sealed blocks. Then, the wax blocks were cut into thin slices and mounted on glass slides. H&E staining was used to assess the pathology of the liver. In addition, oil red O staining was performed on frozen sections of the liver to assess the accumulation of fat in the liver [27].

### 2.10. Western Blots (WB)

Proteins in the mice livers were extracted and quantified. The samples were then added to a polyacrylamide gel, the proteins were separated by electrophoresis and the proteins in the gel were transferred to a PVDF membrane. The membranes were treated with blocking buffer for 2 h to reduce their nonspecific binding. Specific primary antibodies were added and incubated overnight at 4 °C. After washing with TBST solution, the membranes were incubated with the secondary antibody for 2 h. After washing again, the membranes were imaged in a gel imager (Bio−Rad, Hercules, CA, USA) using luminescent solution and analyzed using Image J-1.80 software. The following primary antibodies were used in this study: p−IRS−1 Y^896^ (Abways; AY0440; 1:1000), IRS−1 (Abways, Shanghai, China; CY3428,1:1000), p−PI3K (Abways; CY6428,1:1000), PI3K (Abways; CY6915;1:1000), p−AKT (Abways; CY6569;1:1000), AKT (Abways; CY5551;1:1000), p−GSK3β (Abways; CY6568;1:2000), GSK3β (Abways; CY2434;1:1000), GLUT4 (Bioworld, Beijing, China, MB66704, 1:1000), p−ACC (Abways; CY5094; 1:1000), ACC (Abways; CY5575; 1:1000), MT−ND1 (Abways; CY8176; 1:2000 dilution), SDHB (Abways; CY6860; 1:2000 dilution), UQCRC2 (Abways; CY7110; 1:2000 dilution), COXIV (Abways; CY5155; 1:2000 dilution), TLR4 (Abways; CY5102;1:1000), MyD88 (Abways; CY5681; 1:1000), p−NF−κB (Abways; CY5095; 1:1000 dilution), NF−κB (Abways; CY5034; 1:1000 dilution), β−actin (Abways; AB0011; 1:5000 dilution).

### 2.11. Immunohistochemistry Staining

After the tissue samples were embedded in paraffin, sectioned and dehydrated, they were deparaffinized and hydrated using xylene and graded ethanol. Samples were permeated with Triton−X 100 (0.5%, Beyotime, Shanghai, China) for 15 min and sections were treated with 3% H_2_O_2_ solution to block endogenous peroxidase. Then, the primary antibody (incubated at 4° overnight) and the HRP secondary antibody were incubated successively. Finally, the sections were visualized using diaminobenzidine (DAB). Images were acquired using an inverted fluorescence microscope. The primary antibody used was FAS (Abways, Shanghai, China; CY6600; 1:150).

### 2.12. Real−Time Fluorescence Quantitative PCR (RT−qPCR)

We extracted total RNA from the mouse liver and performed a reverse transcription reaction to synthesize the first strand of cDNA. mRNA levels were quantified by RT−qPCR using a CFX96TM real−time system (Bio−Rad, Hercules, CA, USA). Gene-specific mouse primers were used as shown in Supplementary Table S1. The expression level of the target gene was quantitatively analyzed relative to the 2^−ΔΔCt^ method (*n* = 6/group).

### 2.13. Sequence Analysis of the 16S rRNA Gene of Intestinal Microbiota

Cecal contents were collected from the euthanized mice and submitted to Shanghai Baiqu Biomedical Technology Co., Ltd. (Shanghai, China) for gut microbiota analysis. Fresh fecal samples were collected from mice in each group and stored frozen at −80 °C until DNA extraction. Total fecal microbial DNA was extracted using the MP FastDNA™ Spin Kit for Soil (MP Biomedicals, Santa Ana, CA, USA), and its concentration and purity were determined by agarose gel electrophoresis. Primers 341F (5′-CCTACGGGNGGCWGCAG-3′) and 805R (5′-GACTACHVGGGTATCTAATCC-3′) were used for PCR amplification targeting the V3−V4 hypervariable region of the 16S rRNA gene to construct a sequencing library. Paired-end sequencing was performed on an Illumina NovaSeq 6000 platform (PE250) with the Illumina NovaSeq 6000 S4 Flow Cell (2 × 250 bp, Illumina, San Diego, CA, USA). A bioinformatics pipeline was used for sequencing data processing: raw reads were filtered with Trimmomatic v0.39 (parameters: LEADING:3, TRAILING:3, SLIDINGWINDOW:4:15, MINLEN:150) to remove low−quality, adapter and short reads, and clean reads were quality−verified with FastQC v0.11.9. High−quality reads were clustered into Amplicon Sequence Variants via DADA2 v1.24.0 with default parameters. Taxonomic annotation of 16S rRNA data was performed using QIIME2 v2022.11 with SILVA 138.1 and NT−16s databases to annotate ASVs at all taxonomic levels. Alpha diversity analysis was used to evaluate the richness and evenness of the gut microbiota within each sample. Alpha diversity indices including ASV richness, Chao1 index and Shannon index were calculated using QIIME2 v2022.11. Statistical comparisons among groups were performed using the Kruskal–Wallis test, and *p* < 0.05 was considered statistically significant. Exact *p*−values are presented in the corresponding figures and figure legends. Beta diversity analysis based on principal coordinate analysis (PCoA) was performed to compare differences in microbial community structure among groups.

For the functional prediction of gut microbiota, gene targets corresponding to the significantly different microbiota after SW intervention were obtained from the GutMGene database [28]. Duplicate entries were removed, and the remaining gene targets were regarded as core targets. Protein–protein interaction networks were constructed with a confidence score > 0.9 and nodes with degree > 2. Kyoto Encyclopedia of Genes and Genomes (KEGG) pathway enrichment analysis was performed based on these core targets, and the top 20 KEGG pathways were visualized for further evaluation.

### 2.14. Network Pharmacological Analysis

The metabolomic data of SW were preprocessed (including denoising, normalization and statistical analysis) by MetaboAnalyst 5.0, from which differentially expressed metabolites related to biological activity were extracted, and the detailed information of these metabolites was obtained in PubChem. Then, we entered the chemical structural formula of differences metabolites into SwissTarget Prediction (http://www.swisstargetprediction.ch/) to predict potential targets. Genetic information for T2DM−related genes was retrieved from GeneCard (https://www.genecards.org/) and OMIM (https://omim.org/), merged and duplicated genes were removed. Next, a Venn diagram was used to show the cross network between SW targets and T2DM targets, and the key targets that may improve T2DM were screened out. Cytoscape 3.9.1 was used to construct a network map of active compounds in germinated wheat and T2DM targets. The cross targets between SW and T2DM were imported into STRING (https://cn.string-db.org/) and the interaction score was set to be greater than 0.9 for screening. The common targets obtained from the screening were analyzed by GO and KEGG pathways. To further identify the key targets, MCC, MNC, Degree and Closeness were selected using the “cytoHubba” function of Cytoscape_v3.9.1 [29].

### 2.15. Molecular Docking

The significantly upregulated differential metabolites in SW were docked with the four key gene targets screened. Firstly, the structures of key components of SW were obtained from the PubChem database (PubChem.NCbi.nlm.nih.gov). Crystal structures of key targets were obtained from the RCSB PDB database (https://www.rcsb.org) and Uniprot (https://www.uniprot.org). Molecules and proteins were then docked in AutoDock Vina 1.5.7 after removing water molecules or ligands and hydrogenating, and binding energy was recorded. The docking results with binding energy ≤−6 kcal/mol were visualized by Pymol 2.5.0 software.

### 2.16. Statistical Analysis

All statistical analyses were performed using GraphPad Prism 10 (GraphPad Software, San Diego, CA, USA). Quantitative data are presented as mean ± standard deviation. Intergroup comparisons were conducted using one−way analysis of variance (ANOVA), with post hoc Tukey’s test applied for multiple comparisons. A *p*−value of less than 0.05 was considered statistically significant.

## 3. Results

### 3.1. Impact of Germination on the Nutritional Components and Structural Characterization of Wheat

To preliminarily evaluate the effects of germination on the nutritional components and structural characteristics of wheat, the contents of starch, protein, fat and dietary fiber in unsprouted wheat (USW) and sprouted wheat (SW) were determined. As shown in Figure 1B, compared with USW, the starch content of SW decreased significantly by 15.38% (*p* < 0.05), while the fat content increased significantly by 20% (*p* < 0.05). In contrast, the protein content exhibited a slight but non-significant increase. Although the total dietary fiber content of SW decreased, the soluble dietary fiber (SDF) content increased significantly by 14.94% (*p* < 0.05). Additionally, the total polyphenol and total flavonoid contents in SW were significantly elevated (*p* < 0.05), which was accompanied by a corresponding improvement in antioxidant capacity (Figure 1C,D). These compositional changes are mainly attributed to the enhanced activity of endogenous enzymes (such as amylase, protease and cell wall degrading enzymes) during germination, which facilitates the breakdown of storage substances like starch and promotes the synthesis of secondary metabolites [14].

SEM observations revealed that the surface of USW was tight and intact without obvious cracks, whereas the surface integrity of SW was damaged, with increased intercellular gaps and distinct holes or cracks (Figure 1E). FTIR spectroscopy analysis showed that the absorption peak positions of characteristic functional groups in wheat before and after germination were basically consistent, but the intensities of some characteristic peaks exhibited subtle differences (Figure 1F). As the main component of wheat, starch granules in SW displayed a rough, concave and wrinkled surface compared with those in USW (Figure 1G). During germination, the amorphous region of wheat starch was preferentially hydrolyzed, while the crystalline region was relatively enriched; this preferential hydrolysis ultimately led to a significant increase in starch crystallinity (Figure 1H,I). In summary, these findings indicate that the germination process has had varying degrees of influence on the nutritional components and structural characteristics of wheat.

### 3.2. Untargeted Metabolomics Analysis of Sprouted Wheat

To comprehensively characterize the metabolic differences between USW and SW, untargeted metabolomics was employed to compare their metabolic profiles. Principal component analysis (PCA) and partial least squares discriminant analysis (OPLS−DA) showed clear separation between the two groups, with all sample points distributed within the 95% confidence interval, confirming significant differences in metabolic profiles between USW and SW (Figure 2A,B).

Model validation was performed to evaluate reliability and avoid overfitting: the goodness−of−fit parameter R^2^Y was 0.999, and the predictive ability parameter Q^2^ was 0.998, both approaching 1.0. Additionally, the Q^2^ intercept from permutation test was less than zero, indicating good fit between the model and the actual sample data (Figure 2C). After data preprocessing and annotation, a total of 507 known metabolites were identified in both positive and negative ion modes. Based on their chemical structures and properties, these metabolites were classified into 12 categories, including lipids, phenolic acids, flavonoids, amino acids, organic acids, alkaloids and other classes. A total of 49 significant differential metabolites were identified in the comparison between USW and SW. Among these, 35 metabolites were significantly upregulated and 14 were significantly downregulated in the SW group relative to the USW group (Figure 2D,E). The significantly upregulated metabolites included 4−aminophenol, trans-citric acid, benzyl acetate, L−tartaric acid, ferulic acid and 2−(formylamino)benzoic acid. Notably, these differential metabolites were mainly concentrated in the categories of organic acids, phenolic acids, flavonoids and alkaloids, which are bioactive compounds that may contribute to the functional properties of SW (Figure 2F,G).

### 3.3. The Effect of Sprouted Wheat on the Basic Indicators and Insulin Resistance of T2DM Mice

Based on the compositional and structural changes induced by wheat germination, we hypothesized that SW may possess antidiabetic potential, which was further verified using a mouse model of T2DM. After one week of acclimatization, mice were fed with corresponding diets for 11 consecutive weeks (Figure 3A).

As shown in Figure 3B, body weight began to diverge significantly among the four groups, and the intergroup difference became increasingly pronounced by week 11. Compared with the control group, mice in the HFD and HFD+RW groups exhibited significant increases in body weight, whereas the HFD+SW group showed attenuated body weight gain. Water intake was also significantly reduced in the HFD+SW group, while no significant difference was observed in energy intake among groups (Figure 3C–F). As shown in Figure 3B, body weight began to diverge significantly among the four groups, and the intergroup difference became increasingly pronounced by week 11. Compared with the control group, mice in the HFD and HFD+RW groups exhibited significant increases in body weight, whereas the HFD+SW group showed attenuated body weight gain. Water intake was also significantly reduced in the HFD+SW group, while no significant difference was observed in energy intake among groups (Figure 3C–F). Impaired glucose homeostasis is a hallmark feature of T2DM. To evaluate the effect of SW on glucose metabolism, FBG, OGTT and ITT were performed. SW intervention significantly reduced FBG levels by 35.23% and markedly improved glucose tolerance in long-term HFD−fed mice (Figure 3G,H,J). ITT results further demonstrated that SW significantly enhanced insulin sensitivity in HFD-induced diabetic mice (Figure 3I,K). To explore the underlying molecular mechanism, hepatic protein expression was analyzed by western blotting. The phosphorylation levels of IRS-1 Y896, PI3K, Akt and GSK3β, as well as the protein expression of GLUT4, were significantly downregulated in the HFD and HFD+RW groups. In contrast, these insulin-signaling molecules were markedly restored in the HFD+SW group (Figure 3L,M).

### 3.4. The Effect of Sprouted Wheat on Lipid Metabolism Disorders in T2DM Mice

**Figure 4:**
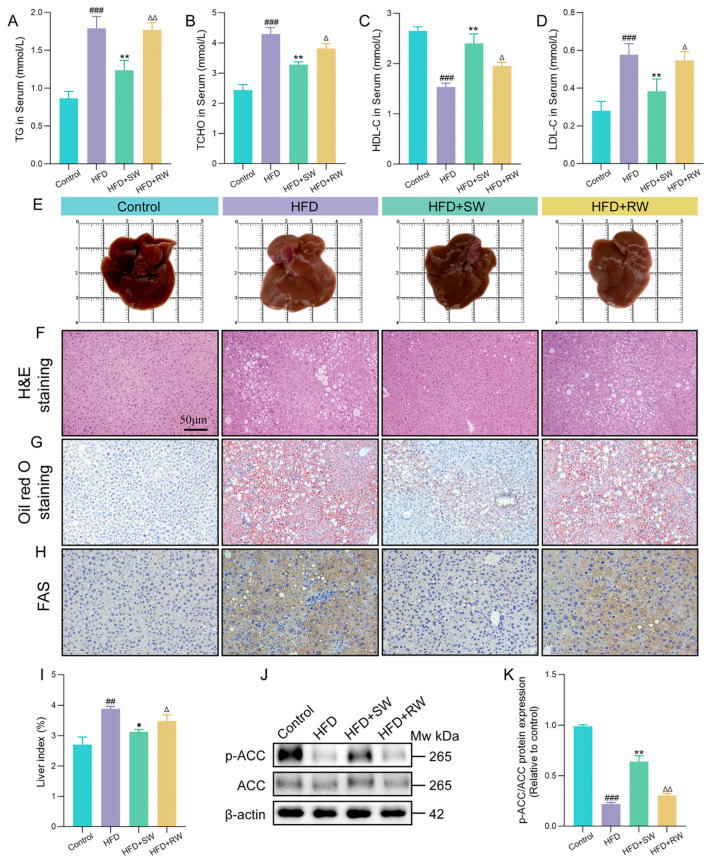
The effect of sprouted wheat on lipid metabolism disorders in T2DM mice. (**A**–**D**) Serum levels of TG, TCHO, HDL−C and LDL−C. (**E**) Liver images. (**F**) H&E staining of the liver in each group. (**G**) Oil red O staining of the livers in each group (lipid droplets). (**H**) IHC staining of FAS expression in livers of each group (positive staining). (**I**) Liver index. (**J**,**K**) The protein expression of p−ACC, ACC and β−actin was used as loading control. Data are presented as mean ± SEM, *n* = 3–6. ^##^ *p* < 0.01, ^###^ *p* < 0.001 versus control group, * *p* < 0.05, ** *p* < 0.01, versus HFD group; ^∆^ *p* < 0.05, ^∆∆^ *p* < 0.01, versus HFD+SW group.

To evaluate the regulatory effect of SW on lipid metabolism in high−fat diet−induced T2DM mice, key serum lipid markers were measured. As shown in Figure 4A–D, compared with the HFD group, the serum levels of TG, TCHO and LDL−C in the HFD+SW group were significantly decreased by 30.7%, 23.6% and 33.4%, respectively (*p* < 0.05), while HDL−C was significantly increased by 56.74% (*p* < 0.05). In contrast, no significant differences in serum lipid profiles were observed between the HFD and HFD+RW groups. Morphologically, mice in the HFD and HFD+RW groups exhibited increased liver volume, yellowish liver color and a significantly elevated liver index compared with the control group (Figure 4E,I). Histological staining revealed that hepatocytes in the control group were closely arranged with normal morphology, whereas hepatocytes in the HFD and HFD+RW groups showed obvious vacuolation and severe lipid droplet accumulation—phenomena that were significantly alleviated in the HFD+SW group (Figure 4F,G). Furthermore, immunohistochemical staining results showed that the expression of FAS was significantly upregulated in the HFD and HFD+RW groups compared with the control group (*p* < 0.05), while SW intervention significantly reversed this upregulation (Figure 4H). At the molecular level, compared with the HFD and HFD+RW groups, the HFD+SW group exhibited significantly increased phosphorylation of ACC (Figure 4J,K). These results indicate that SW intervention effectively improves serum lipid metabolism disorders and alleviates hepatic lipid accumulation in HFD−induced T2DM mice, which is closely associated with its ability to downregulate FAS expression and upregulate ACC phosphorylation, thereby suppressing hepatic fatty acid synthesis.

### 3.5. The Effect of Sprouted Wheat on Mitochondrial Dysfunction in T2DM Mice

**Figure 5:**
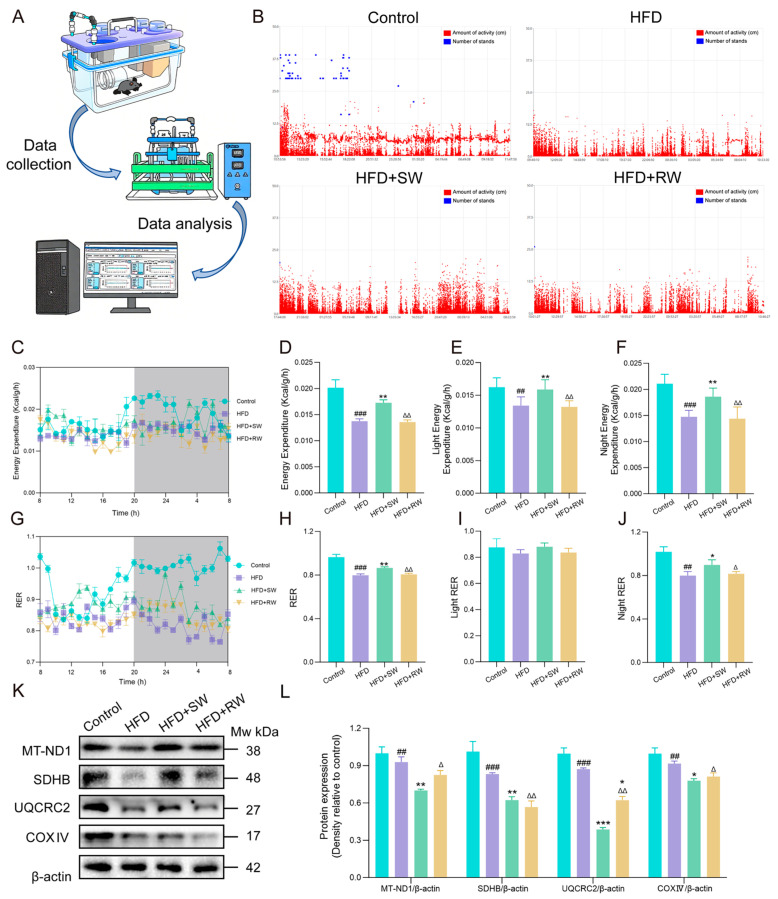
The effect of sprouted wheat on mitochondrial dysfunction in T2DM mice. (**A**) Metabolic cage equipment. (**B**) Scatter plot of 24 h activity and standing of mice. (**C**) The 24 h energy expenditure line graphs of mice in each group. The grey part represents night. (**D**) Bar charts of energy expenditure in each group of mice. (**E**) Bar charts of light energy expenditure of mice in each group. (**F**) Bar charts of night energy expenditure of mice in each group. (**G**) The 24 h RER line graphs of mice in each group. The grey part represents night. (**H**) Bar charts of RER in each group of mice. (**I**) Bar charts of light RER of mice in each group. (**J**) Bar charts of night RER of mice in each group. (**K**,**L**) The protein expression of MT−ND1, SDHB, UQCRC2, COXIV and β−actin was used as loading control. Data are presented as mean ± SEM, *n* = 3–6. ^##^ *p* < 0.01, ^###^ *p* < 0.001 versus Control group, * *p* < 0.05, ** *p* < 0.01, *** *p* < 0.001 versus HFD group; ^∆^ *p* < 0.05, ^∆∆^ *p* < 0.01, versus HFD+SW group.

T2DM may cause mitochondrial dysfunction, resulting in disorders of energy metabolism [30]. To investigate the effect of SW on energy metabolism in high−fat diet−induced T2DM mice, a metabolic cage experiment was conducted to analyze locomotor activity, energy expenditure (EE) and respiratory exchange ratio (RER) in each group (Figure 5A). As shown in Figure 5B–F, locomotor activity and EE were significantly lower in the HFD and HFD+RW groups compared with the control group (*p* < 0.05). Regarding RER, mice in the control group had a RER of approximately 1.0, indicating that their energy was mainly derived from carbohydrates. In contrast, mice in the HFD and HFD+RW groups had a RER between 0.7 and 0.8, suggesting a shift in energy source from carbohydrates to lipids, which is associated with reduced glucose utilization. Notably, the HFD+SW group exhibited significantly increased locomotor activity and EE (*p* < 0.05), with a RER ranging from 0.85 to 0.9. This RER value reflects a mixed energy utilization pattern (both carbohydrates and lipids), which is indicative of enhanced glucose utilization (Figure 5G–J). Between 8:00 AM and 8:00 PM, no significant differences in RER were observed among groups, likely due to the nocturnal nature of mice, which are less active during the daytime. To further evaluate mitochondrial function, we analyzed the expression levels of proteins related to the mitochondrial electron transport chain (ETC) complexes via WB. The WB results showed that the expression levels of MT−ND1, SDHB, UQCRC2 and COXIV were significantly downregulated in the HFD and HFD+RW groups compared with the control group, while SW intervention significantly upregulated their expression (Figure 5K,L).

### 3.6. The Effect of Sprouted Wheat on the Inflammatory Response in T2DM Mice

**Figure 6:**
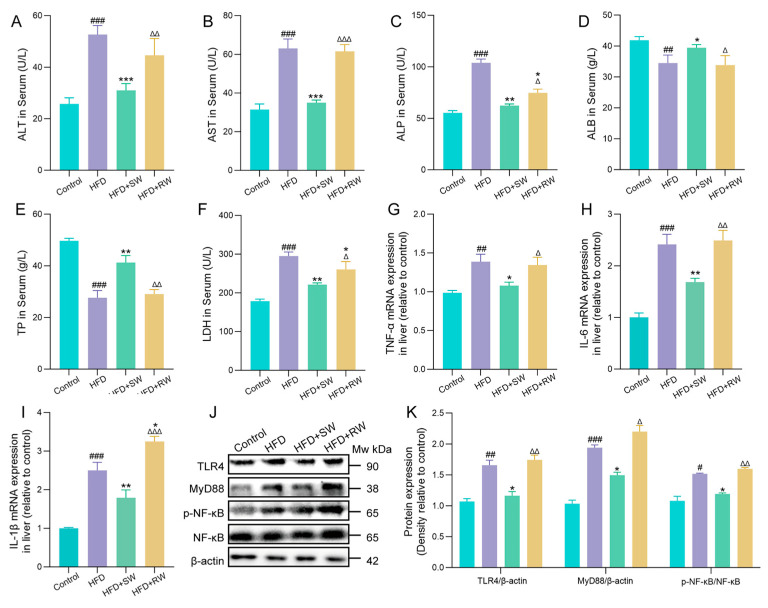
The effect of sprouted wheat on the inflammatory response in T2DM mice. (**A**–**F**) Serum levels of ALT, AST, ALP, ALB, TP and LDH. (**G**–**I**) The mRNA levels of TNF−α, IL−6, IL−1β and GAPDH were used as control. (**J**,**K**) The protein expression of TLR4, MyD88, p−NF−κB/NF−κB and β−actin was used as loading control. Data are presented as mean ± SEM, *n* = 3–6. ^#^ *p* < 0.05, ^##^ *p* < 0.01, ^###^ *p* < 0.001 versus control group, * *p* < 0.05, ** *p* < 0.01, *** *p* < 0.001 versus HFD group; ^∆^ *p* < 0.05, ^∆∆^ *p* < 0.01, ^∆∆∆^ *p* < 0.001 versus HFD+SW group.

A high−fat diet induces excessive ROS production in mitochondria, which may further cause chronic inflammation and liver injury [31]. As shown in Figure 6A–F, compared with the control group, the serum levels of liver function markers (ALP, ALT, AST and LDH) were significantly elevated in the HFD and HFD+RW groups, while the levels of ALB and TP were significantly decreased. These changes indicated that HFD feeding induced obvious liver damage in mice, whereas SW intervention significantly reversed these abnormal liver function indicators (Figure 6A–F). To further explore the anti-inflammatory effect of SW and its underlying mechanism, the expression levels of pro-inflammatory cytokines were detected. Compared with the control group, the serum levels of TNF−α, IL−6 and IL−1β were significantly increased in the HFD and HFD+RW groups, indicating a robust hepatic inflammatory response in these groups. In contrast, SW intervention significantly downregulated the expression of these pro-inflammatory cytokines (Figure 6G–I). We further investigated the TLR4/MyD88/NF−κB signaling pathway. As shown in Figure 6J,K, HFD feeding significantly enhanced TLR4 protein activation and promoted the phosphorylation of NF−κB via the MyD88-dependent pathway, as evidenced by increased TLR4, MyD88 protein expression and NF−κB phosphorylation levels. Notably, SW intervention significantly reduced TLR4 and MyD88 protein expression and inhibited NF−κB phosphorylation.

### 3.7. Sprouted Wheat Modulates Gut Microbiota Composition in T2DM Mice

**Figure 7:**
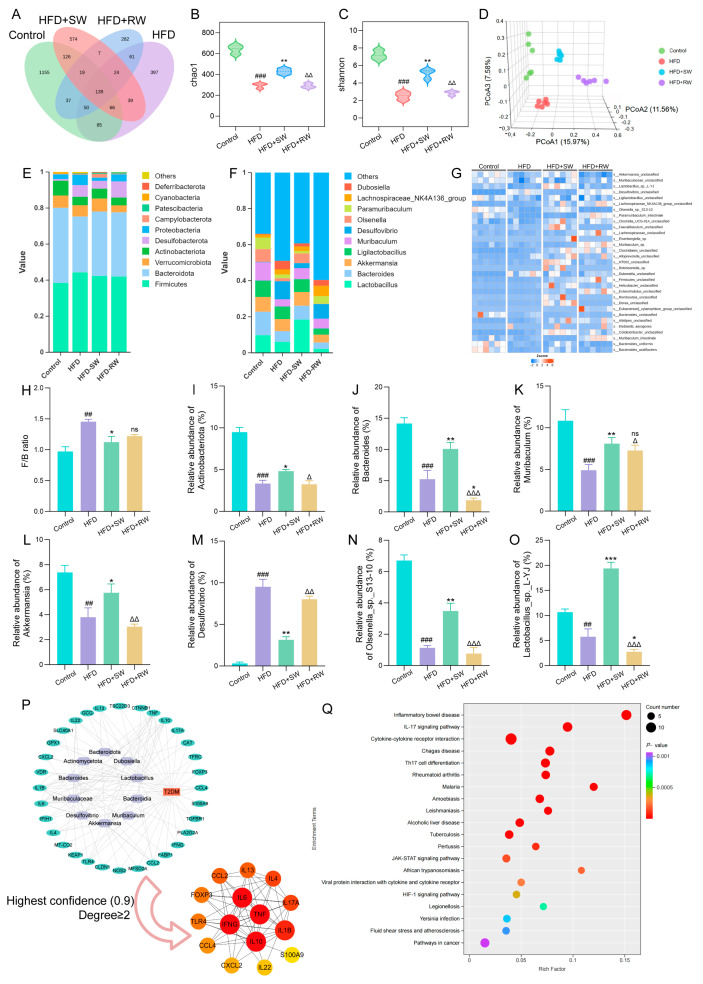
Sprouted wheat modulates gut microbiota composition in T2DM mice. (**A**) Venn diagram. (**B**) Chao1 index. (**C**) Shannon. (**D**) Principal component analysis. (**E**) Stacked plot of phyla−level communities. (**F**) Stacking map of genus−level communities. (**G**) Heat map of species−level communities. (**H**) F/B radio. (**I**–**O**) *Actinobacteriota*, *Bacteroides*, *Muribaculum*, *Akkermansia*, *Desulfovibrio*, *Olsenella_sp._S13−10*, *Lactobacillus_sp._L−YJ* abundance. (**P**) The network diagram of 10 key bacteria and 37 predicted proteins and the interaction network diagram of the selected proteins. (**Q**) KEGG bubble map enriched for 37 predicted proteins (Top 20). *n* = 6., ^##^ *p* < 0.01, ^###^ *p* < 0.001 versus control group, * *p* < 0.05, ** *p* < 0.01, *** *p* < 0.001 versus HFD group; ^∆^ *p* < 0.05, ^∆∆^ *p* < 0.01, ^∆∆∆^ *p* < 0.001 versus HFD+SW group, ns: no significant difference.

The Venn diagram shows the shared and unique ASVs among the different experimental groups. Each circle represents one group. The overlapping regions indicate the number of common ASVs shared between the groups, while the non−overlapping regions represent the number of specific ASVs unique to each group. Different colors are used to distinguish shared and unique ASVs. Compared with the control group, the ASV richness, Chao1 index and Shannon index of the gut microbiota in HFD and HFD+RW groups were significantly decreased (*p* < 0.05). This finding indicates that long-term HFD feeding impairs the richness and diversity of the intestinal microbiota, whereas SW intervention significantly alleviated this declining trend (Figure 7A–C).

Principal coordinate analysis (PCoA) was performed based on the Bray–Curtis distance to reveal differences in gut microbial community structure among the four groups. The first, second and third principal components explained 15.97%, 11.56% and 7.58% of the total variation, respectively. Samples from different groups exhibited distinct separation and good intra−group clustering, indicating that SW intervention effectively reshaped the gut microbiota community structure under HFD conditions (Figure 7D). At the phylum level, *Firmicutes* and *Bacteroidetes* were the dominant phyla in all the groups. Compared with the control group, the HFD and HFD+RW groups exhibited a significantly increased *Firmicutes*/*Bacteroidetes* (F/B) ratio, along with decreased abundances of *Actinobacteria* and *Verrucomicrobia* (*p* < 0.05). SW intervention significantly downregulated the F/B ratio and partially restored the abundances of *Actinobacteria* and *Verrucomicrobia* (Figure 7E,H,I). At the genus level, the abundances of *Bacteroides* and *Akkermansia* were significantly decreased, while the abundance of *Desulfurvibrio* was significantly increased in the HFD and HFD+RW groups compared with the control group (Figure 7F,J–M). SW intervention partially reversed these abnormal changes and significantly increased the abundances of *Olsenella* sp. *S13−10* and *Lactobacillus* sp. *L−YJ* (Figure 7G,N,O). To further explore the mechanism of the intestinal microbiota-mediated protective effect of SW on T2DM, we retrieved 59 microbiota-related target genes from the GutMGene database. These genes overlapped with 37 genes among the top 2000 T2DM-related genes. Protein–protein interaction (PPI) analysis was performed using the STRING database (confidence ≥ 0.9, Degree ≥ 2), leading to the screening of 15 key genes. Among these, TNF and IL−6 ranked as the top two genes based on degree values (Figure 7P). KEGG enrichment analysis showed that the 37 overlapping genes were involved in 52 signaling pathways, among which inflammatory bowel disease and IL−17 signaling pathways were the most significant (Figure 7Q).

### 3.8. Network Pharmacology Analysis of Sprouted Wheat Metabolites and T2DM

**Figure 8:**
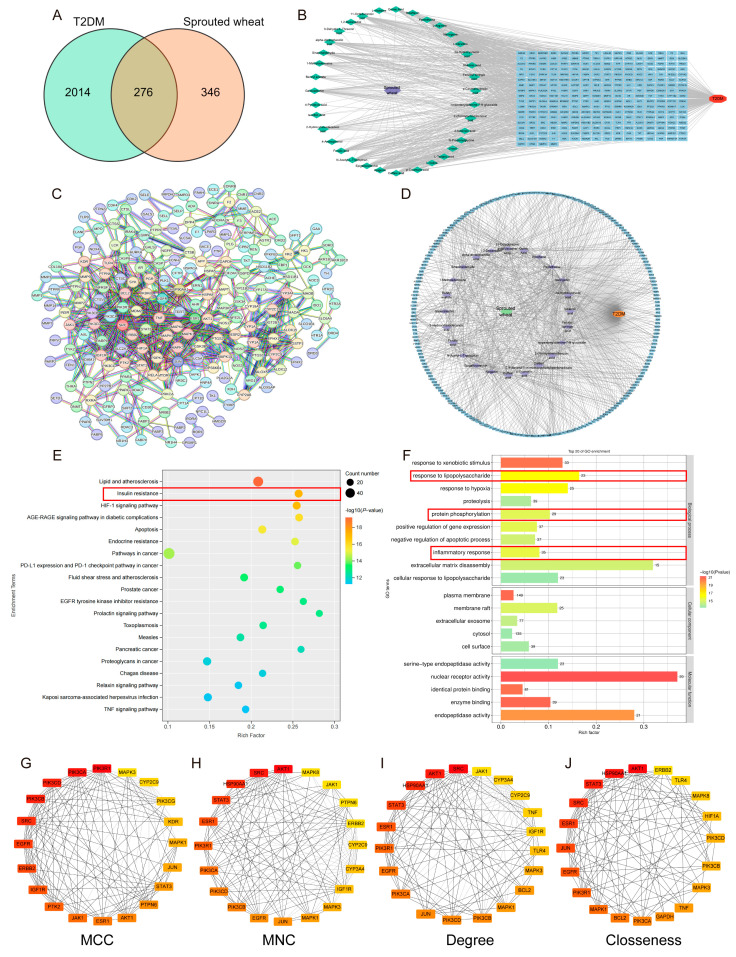
Network pharmacology analysis of sprouted wheat metabolites and T2DM. (**A**) Venn diagram of the targets of SW and T2DM. (**B**) T2DM–target–SW network. (**C**) PPI network. (**D**) T2DM–target–SW network. (**E**) Bubble plot of the potential targets obtained from KEGG enrichment analysis (top 20). (**F**) GO enrichment analysis of the predicted targets in the biological process, molecular function and cell composition categories (top 20). (**G**–**J**) Hub genes were identified using the MCC, MNC, Degree and Closeness algorithms in Cytoscape.

To identify the key bioactive metabolites of SW and their potential gene targets involved in T2DM regulation, network pharmacology analysis was performed. Firstly, metabolites were screened based on fold change (FC) and peak response values, and compounds without predicted targets were excluded. This process identified 24 key metabolites, including citric acid, L–tartaric acid, quinic acid, shikimic acid and pinocembrin. A total of 276 potential gene targets of these metabolites were predicted using the SwissTargetPrediction database. Venn analysis was conducted to intersect the 276 metabolite−related genes with 2000 T2DM−related genes, resulting in 262 potential gene targets associated with both SW metabolites and T2DM (Figure 8A). Based on these 24 metabolites and 262 target genes, a “T2DM–compound–gene target” interaction network was constructed (Figure 8B). To further screen for core gene targets, the confidence threshold was set to the highest level (0.9) using the STRING database, yielding 209 core gene targets that were directly associated with 23 of the 24 metabolites (Figure 8C,D). The 209 core target genes were imported into the DAVID database for KEGG and GO analysis. A total of 180 significantly enriched KEGG pathways were identified, among which the insulin resistance pathway was the most significantly associated with T2DM. In terms of molecular functions, the key genes were associated with nuclear receptor activity and enzyme binding. These functions are closely related to biological processes, including the response to lipopolysaccharide, protein phosphorylation and inflammatory responses (Figure 8E,F). To further confirm the most critical core targets, four topological parameters (MCC, MNC, Degree and Closeness) were used for comprehensive analysis via Cytoscape software. The results showed that PIK3R1, AKT1 and SRC were the top three core target genes, suggesting they play pivotal roles in mediating the regulatory effects of SW metabolites on T2DM (Figure 8G–J). Notably, these three genes are key components of the IRS1/PI3K/AKT signaling pathway, which we previously demonstrated to be regulated by SW in vivo, confirming the consistency between network pharmacology predictions and experimental results.

### 3.9. Molecular Docking Analysis

**Figure 9:**
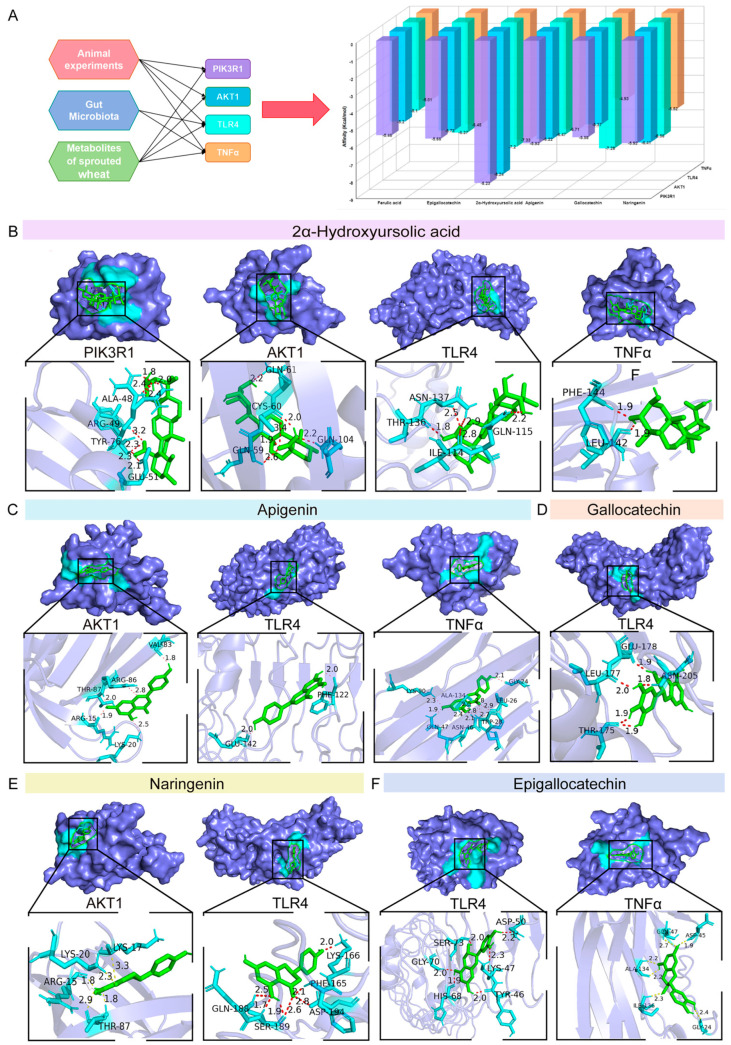
Molecular docking analysis of key metabolites with core protein targets. (**A**) Binding energies of key metabolites to core protein targets. (**B**) 2α−Hydroxyursolic acid-PIK3R1/AKT1/TLR4/TNF−α. (**C**) Apigenin−AKT1/TLR4/TNF−α. (**D**) Gallocatechin−TLR4. (**E**) Naringenin−AKT1/TLR4. (**F**) Epigallocatechin−TLR4/TNF−α.

Combined with the results of the animal experiments, the gut microbiota and metabolites of SW, PIK3R1, AKT1, TLR4 and TNF−α were identified as the core targets in this study, and the key active components of SW were selected for molecular docking verification with the above targets (AKT1, PDB ID: 7MYX; PIK3R1, PDB ID: 3I5R; TNF−α, PDB ID: 6RMJ; TLR4, PDB ID: 2Z65). The results showed that among the active components selected, only 2α−hydroxyursolic acid, apigenin, epigallocatechin, naringenin, gallocatechin and ferulic acid had binding energies ≤−5 kcal/mol with the above four targets (Figure 9A).

The analysis of pairs with binding energies ≤ −6 kcal/mol indicated that 2α−hydroxyursolic acid had a strong binding ability with all four targets. It formed hydrogen bond interactions with the amino acid residues CYS60 and GLN104 of AKT1 and ARG49 of PIK3R1 and had the same effect as SER71 of TLR4 and PHE144 of TNF−α (Figure 9B). Additionally, apigenin was relatively stable in binding with AKT1, TLR4 and TNF−α, especially when establishing hydrogen bond interactions with TRP28 and GLN47 of TNF−α (Figure 9C). On the other hand, epigallocatechin, naringenin and gallocatechin had stronger binding abilities with inflammation−related TLR4 and TNF−α, suggesting that they may exert biological effects by targeting and regulating the inflammatory pathways (Figure 9D–F).

## 4. Discussion

The germinated grains have significantly enhanced effects on improving glucose and lipid metabolism, regulating intestinal flora and reducing inflammatory response [10,32]. However, the potential health components within it have not been fully explored. With the advantages of integrating multiple research methods, focusing on the microbiota−liver axis (a key pathway related to T2DM) and identifying specific active substances and targets, this study, based on the comprehensive analysis of 16sRNA sequence analysis, untargeted metabolomics and network pharmacology, demonstrated for the first time that SW ameliorates metabolic disorders and inflammatory responses in the liver of T2DM mice by regulating the microbiota−liver axis mechanism. In this process, the five substances of 2α−hydroxyursolic acid, apigenin, epigallocatechin, naringenin, gallocatechin and the four core targets of PIK3R1, AKT1, TLR4 and TNF−α play a more critical role.

T2DM, as a prevalent chronic metabolic disease worldwide, is closely related to metabolic disorders, chronic inflammation and insulin resistance caused by a long−term high-calorie diet [33,34,35]. Previous studies have shown that a whole−grain diet can prevent or delay metabolic diseases such as diabetes by utilizing its rich bioactive substances (such as dietary polysaccharides, flavonoids and phenolic acids), which improve glucose and lipid metabolism and reduce inflammation [36,37,38]. To explore whether germination further optimizes the metabolic health benefits of wheat, we preliminarily compared the nutritional composition of sprouted and unsprouted wheat, focusing on components relevant to the management of T2DM. The activation of endogenous hydrolytic enzymes, including amylases and cell wall−degrading enzymes, preferentially decomposes starch granules and cell wall structures in the endosperm. This directly accounts for the significant reduction in starch content and simultaneously leads to microstructural changes, including rough and concave surfaces of starch granules, as well as distinct pores and gaps between cells. Conversely, such structural disruption creates favorable conditions for further component transformation. The loosened cell structure increases the contact area between enzymes and substrates, accelerating the hydrolysis and conversion of reserve substances such as starch and fat. This not only elevates the contents of soluble dietary fiber and small−molecule nutrients (e.g., polyphenols), but also results in the preferential degradation of the amorphous region of starch, thereby leading to a relative increase in its crystallinity [39].

Notably, germination induced differentiated changes in wheat nutrients, which are closely associated with germination−induced structural alterations and jointly enhance its metabolic regulatory function: specifically, starch content decreased by 15.38%, accompanied by structural changes (preferential hydrolysis of starch amorphous regions and increased crystallinity) which slow carbohydrate digestion and absorption to potentially reduce glycemic load. At the same time, SDF content increased by 14.94%, a bioactive ingredient capable of improving glucose homeostasis by delaying gastric emptying, enhancing insulin responsiveness, and inhibiting inflammatory pathways [40,41]. What is more worthy of attention is that germination significantly increases bioactive components (polyphenols, flavonoids and alkaloids), facilitated by structural damage that breaks cell walls and accelerates their release. These components can further enhance their regulatory effect on metabolic disorders by acting through multiple targets, such as inhibiting inflammatory pathways, regulating the balance of intestinal flora structure and improving glycolipid metabolism disorders. These factors are closely related to the development of T2DM, suggesting that the germination process may make the nutritional properties of wheat more conducive to metabolic regulation. Our animal experiments also showed that SW supplementation improved the elevation of fasting glucose levels, insulin resistance, ETC damage and inflammatory response in the liver in both the HFD and HFD+RW groups. This suggests a unique role of SW in ameliorating chronic metabolic disorders.

In addition, we found that SW could effectively improve gut microbiota imbalance in HFD-induced T2DM mice. *Firmicutes* and *Bacteroidetes* are the dominant phylum of intestinal flora, and the ratio of *Firmicutes* to *Bacteroidetes* (F/B) is the core index to evaluate the imbalance of intestinal flora [42]. The results showed that the F/B ratio of the HFD group was significantly increased, while this trend was not significant in the HFD+SW group and the HFD+RW group. *Firmicutes* are closely related to high-calorie, protein, fat and sugar intake, and have higher metabolic efficiency. *Bacteroidetes*, which are associated with dietary fiber intake, have a genome rich in carbohydrate-active enzyme genes that efficiently metabolize polysaccharides such as β−glucan and produce short-chain fatty acids, thereby reducing inflammation and cholesterol levels [43]. SW significantly reversed the reduction in *Bacteroides* levels induced by the HFD, providing a microbial foundation for the production of short−chain fatty acids. Among the *Firmicutes* phylum, the most significantly changed genus in the HFD+SW group was *Lactobacillus*, especially *s_Lactobacillus_sp._L−YJ*. Previous studies have shown that *Lactobacillus* can inhibit the proliferation of pathogenic bacteria by releasing antimicrobial mediators, enhance intestinal barrier function and regulate immune responses, thereby improving metabolic disorders [44,45]. Furthermore, the addition of SW significantly increased the abundance of *Actinobacteria* and *Verrucomicrobia* while reducing the level of *Desulfuribacter*. Through analysis using the gutMgene database, it was found that the functional genes of these bacterial groups can significantly regulate inflammatory pathways. Among them, TNF and IL−6 are the key targets, indicating that SW may inhibit the inflammatory response by reshaping the structure of the intestinal microbiota.

The structure of gut microbiota is regulated by dietary patterns. In addition to dietary fiber, the germination process of SW is also significantly enriched in bioactive compounds such as polyphenols, alkaloids, lignin and vitamins, which are important material bases for regulating gut microbiota. By combining network pharmacology and molecular docking analysis, five key metabolites were screened out: 2α−hydroxyursolic acid, apigenin, epigallocatechin, naringenin and epicatechin. Some of these metabolites have been proven in studies to be able to improve metabolic−related diseases by regulating the intestinal microbiota: naringenin can increase energy consumption by reshaping the gut microbiota, alleviate obesity induced by a HFD and its microbial metabolites can lower total cholesterol and triglyceride levels [46,47]; apigenin alleviates obesity−related low−grade inflammation and insulin resistance by regulating the composition of the intestinal microbiota. This study hypothesizes that these metabolites in SW may reshape the structure of the gut microbiota through a synergistic effect, thereby improving insulin resistance and inflammatory responses in T2DM mice, providing a material basis for the molecular mechanism of SW regulating the intestinal microbiota.

In order to further explore the active ingredients and key mechanisms in SW, we identified five key metabolites (2α−hydroxyursolic acid, apigenin, epigallocatechin, naringenin and gallocatechin), and four core targets, PIK3R1, AKT1, TLR4 and TNF−α, in SW by multi−technology integrated analysis. PIK3R1 and AKT1 are the core nodes of the PI3K/AKT signaling pathway, which is the central pathway of insulin regulation of glucose metabolism. Studies have confirmed that apigenin and naringenin can improve insulin resistance by activating the IRS1/PI3K/AKT signaling pathway [48]. TLR4 is the main receptor for sensing endotoxin (lipopolysaccharide), and the TNF−α produced downstream is a core inflammatory factor that interferes with insulin signaling [49,50]. Previous studies have confirmed that apigenin is an effective inhibitor of the TLR4 signaling pathway, while gallocatechin and epigallocatechin can significantly inhibit the production and release of TNF−α [51,52,53]. In addition, the imbalance of the gut microbiota can damage the intestinal barrier, allowing lipotoxins to enter the bloodstream and triggering systemic inflammation [54]. However, the three may not act in isolation, but they are synergetic through metabolite-mediated bidirectional regulation of microbiota and targets. On the one hand, these metabolites are directly targeted to the liver through the blood to activate the IRS1/PI3K/AKT pathway by binding with high affinity to PIK3R1/AKT1 and are precisely embedded in the TLR4/TNF−α active pocket to directly inhibit inflammatory cytokine release and signaling. The vicious cycle of insulin resistance and inflammation is doubly blocked. On the other hand, the metabolites such as naringenin and apigenin in SW can directly reconstitute the structure of intestinal flora, increase the abundance of beneficial bacteria, reduce endotoxin production, repair the intestinal barrier to block lipotoxicity from entering the blood and weaken the upstream signal of TLR4/NF−κB inflammatory pathway activation from the source.

This study has several limitations: the direct causal link between gut microbiota remodeling and inflammation remains unconfirmed, the intervention duration was short and the specific molecular mechanism of key metabolites regulating core targets needs further clarification. Future research will focus on addressing these limitations, including conducting fecal microbiota transplantation experiments to confirm causal links, performing long−term animal intervention experiments to verify safety, implementing randomized controlled clinical trials in T2DM patients and conducting in vitro human cell experiments to clarify molecular mechanisms, as well as developing SW-derived functional foods to promote its practical application in human T2DM prevention and adjuvant treatment.

## 5. Conclusions

This study clearly clarifies the core molecular mechanisms and material basis of sprouted wheat (SW) in improving hepatic metabolic disorders and inflammatory responses in T2DM mice through the integration of multiple technologies (16sRNA sequence analysis, untargeted metabolomics, network pharmacology and animal experiments) and systematic verification. Specifically, SW exerts its protective effects by reshaping gut microbiota structure, suppressing the TLR4/NF−κB inflammatory pathway and regulating five key bioactive metabolites (2α−hydroxyursolic acid, apigenin, epigallocatechin, naringenin, gallocatechin) and four core gene targets (PIK3R1, AKT1, TLR4, TNF−α). These findings highlight the potential of SW as a natural, non-pharmacological nutritional intervention for T2DM, with clear implications for improving human metabolic health by targeting the gut−liver axis, regulating glucose and lipid metabolism and alleviating chronic inflammation.

## Data Availability

The original contributions presented in this study are included in the article/Supplementary Materials. Further inquiries can be directed to the corresponding authors.

## Supplementary Materials

The following supporting information can be downloaded at: https://www.mdpi.com/article/10.3390/foods15061027/s1, Table S1: Primers used in the experiment and their sequences.

## Abbreviations

The following abbreviations are used in this manuscript:

p−IRS 1 Y^896^	Phosphorylation of insulin receptor substrate 1 at tyrosine 896
IRS 1	insulin receptor substrate 1
p−PI3K	Phosphorylation of phosphatidylinositol 3−Kinase
PI3K	Phosphatidylinositol 3−Kinase
p−AKT	Phosphorylation of protein Kinase B
AKT	Protein Kinase B
p−GSK3β	Phosphorylation of glycogen Synthase Kinase 3β
GSK3β	Glycogen Synthase Kinase 3β
GLUT4	Glucose Transporter 4
p−ACC	Phosphorylation of acetyl-coa carboxylase
ACC	Acetyl−coa carboxylase
FAS	Fatty acid synthase
TLR4	Toll−like receptor 4
MyD88	Myeloid differentiation primary response 88
p−NF−κB	Phosphorylated nuclear factor kappa−B
NF−κB	nuclear factor kappa−B
TNF−α	Tumor necrosis factor−α
IL−6	Interleukin−6
IL−1β	Interleukin−1β
MT−ND1	NADH dehydrogenase 1 Gene
SDHB	Succinate dehydrogenase complex subunit B
UQCRC2	Ubiquinol-cytochrome c reductase core protein II
COXIV	Cytochrome c oxidase 4
PIK3R1	Phosphoinositide−3−kinase, regulatory subunit 1
PVDF	Polyvinylidene fluoride
RT−qPCR	Real−Time quantitative PCR
GAPDH	Glyceraldehyde−3−phosphate dehydrogenase
