# Supplementary material for: Sprouted Wheat Improves Liver Metabolism and Inflammation in T2DM Mice: 16S rRNA Gene Sequence, Metabolomics and Network Pharmacology Joint Analysis
Source: Foods. 2026 Mar 15;15(6):1027. doi: 10.3390/foods15061027 (PMC13025019; doi:10.3390/foods15061027)
Supplement: Supplementary file 1 [file foods-15-01027-s001.zip › Supplementary Material.pdf]

Supplementary Information for

Sprouted Wheat Improves Liver Metabolism and Inflammation in T2DM Mice: 16S rRNA gene sequence, Metabolomics and Network Pharmacology Joint Analysis

Supplementary Methods

Analysis of the structural characteristics of wheat samples and their starches

(1) Scanning electron microscope (SEM) analysis

The wheat samples and starch samples were analyzed by SEM (GeminiSEM 3000, Zeiss, Germany). During SEM testing, the samples were fixed on the sample stage and sprayed with gold, placed under the scanning electron microscope, and the microscopic morphology was observed and the images were collected by setting 5kV acceleration voltage.

(2) Fourier transform infrared spectroscopy (FTIR) analysis

In FTIR measurement, potassium bromide was mixed with potassium bromide at the ratio of 1: 100 and ground uniformly, and then pressed into a Fourier transform infrared spectrometer. Scanning in the wavenumber range of 4000-400 cm, the infrared spectra were recorded to analyze the characteristics of functional groups.

(3) X-ray diffraction (XRD) analysis

XRD analysis of wheat starch was performed using a Bruker D8 Advance X-ray diffractometer with a Cu target. The diffraction parameters were set as follows: 2θ scanning range of 5° to 40° and scanning speed of 4° per minute. Prior to testing, the sample was uniformly spread on the sample holder and compacted to ensure a flat surface. After the test, the crystallinity and crystal structure of wheat starch were systematically evaluated based on the peak shape characteristics and relative intensities of the diffraction pattern.

Supplementary Table S1: Primers used in the experiment and their sequences

**Gene**	**Forward primer (5′ -3′)**	**Reverse primer (5′ -3′)**
GAPDH	5′-AGGTCGGTGTGAACGGATTTG-3′	5′-GGGGTCGTTGATGGCAACA-3′
IL-6	5′-CTGCAAGAGACTTCCATCCAG-3′	5′-AGTGGTATAGACAGGTCTGTTGG-3′
IL-1β	5′-CAGGCGGTGCCTATGTCTCTC-3′	5′-CGATCACCCCGAAGTTCAGTAG-3′
TNF-α	5′-CAGGCGGTGCCTATGTCTC-3′	5′-CGATCACCCCGAAGTTCAGTAG-3′
